# Traits of narcissistic vulnerability in adults with Autism Spectrum Disorders without intellectual disabilities

**DOI:** 10.1192/j.eurpsy.2024.185

**Published:** 2024-08-27

**Authors:** G. Broglia, V. Nistico’, B. Di Paolo, R. Faggioli, A. Bertani, O. Gambini, B. Demartini

**Affiliations:** ^1^Health Science Department, University of Milan; ^2^Department of Psychology, University of Milan Bicocca; ^3^Aldo Ravelli Research Center for Neurotechnology and Experimental Brain Therapeutics, University of Milan; ^4^UO Psichiatria 51 e 52; ^5^Centro Giovani Ettore Ponti, ASST Santi Paolo e Carlo, Presidio San Paolo, Milan, Italy

## Abstract

**Introduction:**

The relationship between Autism Spectrum Disorders (ASD) and Narcissistic Personality Disorder (NPD), considering the dimensions of narcissistic grandiosity and vulnerability, represents an important differential diagnosis and potential ground of comorbidity, since both conditions show high grades of pervasiveness, a life-long course, ego-syntonic traits, and difficulties in building up and sustaining interpersonal relationships Although the co-diagnosis rates, according to the categorical criteria in use, are limited (0%-6.4%), it is common to encounter diagnostic doubts in clinical practice.

**Objectives:**

Here we aimed to explore both the dimensions of narcissistic vulnerability and grandiosity in a group of adults diagnosed with ASD without intellectual disabilities.

**Methods:**

87 individuals with ASD completed the Pathological Narcissism Inventory-52 Items (PNI-52). The mean scores of our sample were compared with the normative distribution available in the literature. Participants also underwent a detailed sociodemographic and anamnestic interview, along with an assessment for autistic traits, comprising the “Ritvo Autism and Asperger Diagnostic Scale-Revised” (RAADS-R) and the Autism Quotient (AQ).

**Results:**

Individuals with ASD scored significantly higher than neurotypical controls at the Total Score and at the Vulnerable Narcissism subscale, but not at the Grandiose Narcissism subscales. Demographic features did not influence these results. Vulnerable narcissism was significantly associated with the RAADS-R subscale Social Relatedness.

**Conclusions:**

Our findings could potentially be indicative of a greater comorbidity rate between the two disorders with respect to the one reported to date, possibly because DSM-5 criteria are mainly focused on the grandiose dimension. Potential explanatory links between ASD phenomenology and vulnerable narcissism, such as the personality dimension of neuroticism, are discussed, together with the possible role of narcissistic vulnerability in mediating internalizing symptoms (e.g., anxiety, depression) in individuals with ASD.

**Disclosure of Interest:**

None Declared

